# The relationship between inflammatory bowel disease and sarcopenia-related traits: a bidirectional two-sample mendelian randomization study

**DOI:** 10.3389/fendo.2024.1402551

**Published:** 2024-07-12

**Authors:** Zhihuang Sun, Guangwei Liu, Jiajia Xu, Xianyu Zhang, Huahua Wei, Guobao Wu, Jian Jiang

**Affiliations:** ^1^ Department of Orthopedics, Shangrao People’s Hospital, Shangrao, China; ^2^ Department of Hematology, Shangrao People’s Hospital, Shangrao, China

**Keywords:** inflammatory bowel disease, ulcerative colitis, Crohn’s disease, sarcopenia-related traits, Mendelian randomization

## Abstract

**Objective:**

Observational studies have revealed a link between inflammatory bowel disease (IBD) and sarcopenia. However, it remains unclear whether this correlation between IBD and sarcopenia is causal.

**Methods:**

The genetic instrumental variables (IVs) associated with IBD and sarcopenia-related traits were derived from publicly available genome-wide association studies. We employed a two-sample bidirectional Mendelian randomization (MR) method. we obtained genetic IVs for five phenotypes from 34,652 cases in IBD, 27,432 cases in ulcerative colitis (UC), 212356 cases in crohn’s disease (CD), 9336415 cases in low hand grip strength (LHGS), and 450243 cases in appendicular lean mass (ALM), respectively. The inverse variance weighting and other MR methods were used to explore the bidirectional causal relationship. Furthermore, we performed heterogeneity test, pleiotropy test, leave-one-out sensitivity test, and multivariate MR to evaluate the robustness of the results.

**Results:**

The forward MR results showed that the UC (OR=0.994, 95% CI: 0.9876–0.9998, P = 0.044) and CD (OR=0.993, 95% CI: 0.988–0.998, P = 0.006) was negatively correlated with ALM. In the reverse MR analysis, we also found that LHGS was negatively correlated with the IBD (OR=0.76, 95% CI: 0.61–0.94, P = 0.012) and CD (OR=0.53, 95% CI: 0.40–0.70, P <0.001). Besides, genetically predicted higher ALM reduced IBD (OR=0.87, 95% CI: 0.79–0.95, P = 0.002), UC (OR=0.84, 95% CI: 0.76–0.93, P = 0.001), and CD (OR=0.87, 95% CI: 0.77–0.99, P = 0.029). However, the results of other MR Analyses were not statistically different.

**Conclusions:**

We found genetically predicted UC and CD are causally associated with reduced ALM, and higher hand grip strength reduced IBD and CD risk, and higher ALM reduced IBDs risk. This MR study provides moderate evidence for a bidirectional causal relationship between IBD and sarcopenia.

## 1 Introduction

According to the latest 2019 European Working Group on Sarcopenia in Older People (EWGSOP) and International Working Group on Sarcopenia (IWGS) (1), sarcopenia is a clinical syndrome characterized by the decline in the muscle mass and/or strength of the whole body, resulting in reduced physical function and quality of life, and heightened susceptibility to physical disability and mortality. It is estimated that sarcopenia affects approximately 10% to 16% of older individuals globally (2), and is linked to a range of unfavorable health consequences, including fractures, reduced function, and mortality (3). In addition to being common in the elderly, it can also develop in middle age (3) and is prevalent in certain high-risk groups, such as cancer patients (4), renal insufficiency (5), liver disease (6), and metabolic disorders (7). Therefore, identifying the causes of sarcopenia and associated risk factors is critical.

Inflammatory bowel disease (IBD) is a chronic, idiopathic inflammatory disorder affecting the gastrointestinal tract, encompassing two primary classifications: Crohn’s disease (CD) and ulcerative colitis (UC). CD has the potential to affect any segment of the gastrointestinal system, ranging from the oral cavity to the perianal region, while UC is distinguished by the presence of widespread and persistent inflammation in the colon, extending from the proximal portion of the rectum (8). The utilization of whole genome sequencing and other genetic research techniques has led to the identification of over 200 sites that are related with the risk of IBD (9, 10). Meanwhile, there has been a notable rise in the prevalence of IBDs in the newly industrialized nations of the twenty-first century (11). Although the precise origins of these events remain uncertain, it can be attributed to a multifaceted interaction between hereditary factors and environmental influences (12, 13). Several recent observational studies have examined the correlation between IBD and sarcopenia. Fatani et al. (14) found that one-fifth of IBD patients had sarcopenia, which was significantly associated with IBD treatment failure and postoperative complications. Nam et al. (15) demonstrated a significant prognostic value between sarcopenia and perianal Crohn’s disease treatment. Mulinacci et al. (16) also showed that the prevalence of sarcopenia in patients with IBD can be as high as 50%, and sarcopenia is associated with a number of adverse clinical outcomes, including higher morbidity, longer hospital stays, increased postoperative complications, and treatment failure. Considering that some patients are concerned that regular physical activity is associated with a potential increase in IBD activity (17), it is still controversial whether it is worthwhile to recommend exercise as an intervention for IBD-related diseases.

Nevertheless, it is unavoidable that observational studies would be subject to bias resulting from confounding effects and reverse causality. Hence, the potential causal connection between IBD and sarcopenia remains uncertain, highlighting the need for further research to confirm this association (18). Therefore, the aim of this study was to perform a two-sample Mendelian randomization (MR) Study design to investigate the potential bidirectional causality between genetically predicted IBD and sarcopenia by performing an updated genome-wide association studies (GWAS) meta-analysis of IBD and sarcopenia.

## 2 Materials and methods

### 2.1 Study design

**Figure 1:**
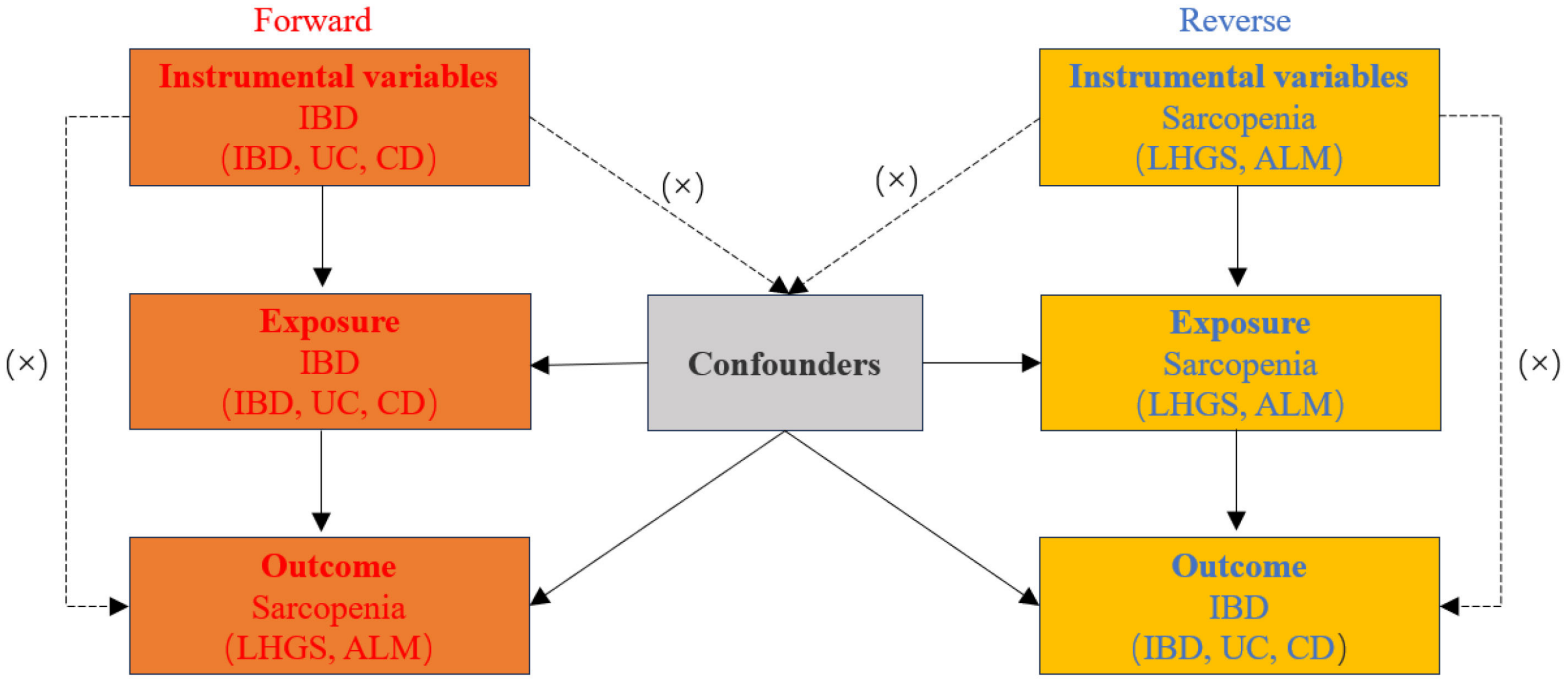
Diagram for three main assumptions of MR study. Lines with arrows indicate that the instrumental variables (IVs) are associated with the exposure and could only affect the outcome through the exposure. Dashed lines show that the IVs are independent of any confounding variables. IBD, inflammatory bowel disease; UC, ulcerative colitis; CD, crohn’s disease; LHGS, low hand grip strength; ALM, appendicular lean mass.

A two-sample MR analysis was used to evaluate bidirectional causation between IBD (including UC and CD subtypes) and sarcopenia-related trait, namely low hand-grip strength (LHGS) and appendicular lean mass (ALM). For this investigation, we chose phenotypic single-nucleotide polymorphisms (SNPs) as genetic instrumental variables (IVs). Meanwhile, the genetic IVs for phenotypes should satisfy the following three key assumptions (19) (**Figure 1**): 1. IVs should be substantially related to exposure; 2. IVs should be unaffected by any possible confounders that may influence the link between the exposure and outcome; 3. IVs should exclusively effect the outcome through exposure. In this work, ethical approval was deemed unnecessary as we used publicly accessible GWAS findings from the IIBDGC database and UK Biobank (20).

### 2.2 Data sources

Summary statistics for IBDs were extracted from the International Inflammatory Bowel Disease Genetics Consortium (IIBDGC). The study of IBDs involved 34,652 cases of European ancestry where the subgroup UC comprised 5,956 patients and 14,927 controls, while the CD subgroup contained 6,968 patients and 20,464 controls (10). According to European Crohn’s and Colitis Organisation, the diagnosis of CD involves a comprehensive evaluation including endoscopic, histopathological, and imaging findings to detect granulomatous inflammation and transmural lesions. The diagnosis of UC primarily relies on continuous colonic involvement and superficial inflammation, supported by histopathological findings, such as crypt architectural changes and mucosal inflammatory infiltrates (21). For IVs of sarcopenia, we selected the latest and largest available GWAS studies. The pooled data for LHGS obtained from a comprehensive meta-analysis GWAS that encompassed a substantial sample size of 256,523 individuals of European descent (22). The cut-off for LHGS was male <30 kg and female <20kg. Summary statistics for ALM were derived from 450,243 individuals in the UK Biobank study (23). Lean soft tissue mass was assessed using Dual-Energy X-ray Absorptiometry (DEXA). ALM was obtained by the sum of upper and lower limbs muscle mass. Exclusions for LHGS and ALM include pregnant women, people who are bedridden, amputees, and people who are unable to take anthropometry or body composition measurements (24).

The GWAS data utilized in this study were obtained exclusively from the IEU OpenGWAS online database, which provides extensive genetic data (ID: ieu-a-31for IBD, ieu-a-30 for CD, ieu-a-32 for UC, ebi-a-GCST90007526 for LHGS, and ebi-a-GCST90000025 for ALM) (**Table 1**). The utilization of data was authorized by the ethics committee of each participating center or nation involved, and all participants provided written, informed permission (10).

**Table 1:** Summary statistics of data source.

Phenotype	First author or Consortium	Sample size	No. of patients	No. of controls	No. of variants	Population	Trait ID GWAS	Year
IBD	IIBDGC	34,652	12,882	21,770	12,716,084	European	ieu-a-31	2015
CD	IIBDGC	27,432	6,968	20,464	12,255,197	European	ieu-a-32	2015
UC	IIBDGC	20,883	5,956	14,927	12,276,506	European	ieu-a-30	2015
LHGS	Jones G	256,523	48,596	207,927	9,336,415	European	ebi-a-GCST90007526	2021
ALM	Pei YF	450,243	NA	NA	18,071,518	European	ebi-a-GCST90000025	2020

IIBDGC, The International Inflammatory Bowel Disease Genetics Consortium; IBD, Inflammatory bowel disease; UC, Ulcerative colitis; CD, Crohn’s disease; LHGS, Low hand grip strength; ALM, appendicular lean mass; NA, not available.

### 2.3 Instruments selection

In this study, we identified single-nucleotide polymorphisms (SNPs) that were strongly associated with IBD, UC, CD, LHGS and ALM, with genome-wide significance of P < 5 × 10^–8^. To ensure independence, IVs were subjected to a PLINK clustering process. To eliminate SNPs associated with considerable linkage disequilibrium (LD), we employed a clustering method with r^2^ < 0.001 and a clumping distance of 10,000 kb. We then evaluated the strength of the IVs using a F statistic greater than 10 to reduce the influence of feeble IVs on the causal analysis. The F statistic is computed using the formula outlined in the prior literature (25). To avoid potential pleiotropy, IVs associated with confounding or risk factors for sarcopenia (older age, low socioeconomic status, low physical activity, and inadequate diet) were excluded using the PhenoScanner V2 (http://www.phenoscanner.medschl.cam.ac.uk/) (3).

Lastly, SNPs excluding confounding variables were utilized for subsequent MR analysis. These IVs are described in detail in **Supplementary Tables 1**–**5**, and all confounders in sarcopenia and excluded SNPs are listed in **Supplementary Table 6**.

### 2.4 Mendelian randomization analyses

In the MR analysis, multiple statistical methodologies were combined. The primary method was the inverse variance weighted (IVW), which is expected to be stable due to its balanced pleiotropy. For supplementary and substitution analysis, weighted mode, simple mode, weighted median, and MR Egger methods were utilized concurrently. Furthermore, the MR-PRESSO (MR pleiotropy residual sum and outlier) test was performed to correct for potential confounding variables (26).

### 2.5 Sensitivity analysis

Horizontal pleiotropy occurs when IVs associated with the exposure influence the outcome via multiple factors besides the exposure. We used the MR-Egger intercept test in MR-Egger regression to determine the presence of horizontal pleiotropy. A significant intercept (P < 0.05) indicates the presence of pleiotropy, and the results should be interpreted with caution. The scatter plots are utilized to display the results of the MR-Egger intercept test. We also used Cochran’s Q statistics to investigate heterogeneity, where significant heterogeneity (P < 0.05) indicates the presence of heterogeneity among the included SNPs, and the random effects MR analysis was used as the primary analysis technique. Moreover, funnel plots were used to visualize the heterogeneity results. We use the MR-PRESSO outlier test to remove aberrant SNPs (outliers) and estimate the corrected results in order to eliminate horizontal pleiotropy. In the leave-one-out analysis, results were reanalyzed after removing one SNP at a time, and forest plots were drawn to assess the stability of the results intuitively.

### 2.6 Statistical analysis and data visualization

The “TwoSampleMR” and “MR-PRESSO” packages of the R software (version 4.2.2) were employed for estimating causal effects and identifying outliers. Results are presented as an odds ratio (OR) with a 95% confidence interval (CI). P < 0.05 is considered to have a significant difference.

## 3 Results

### 3.1 Causal effects of IBD on Sarcopenia-related traits

#### 3.1.1 Causal effects of IBD on LHGS

**Figure 2:**
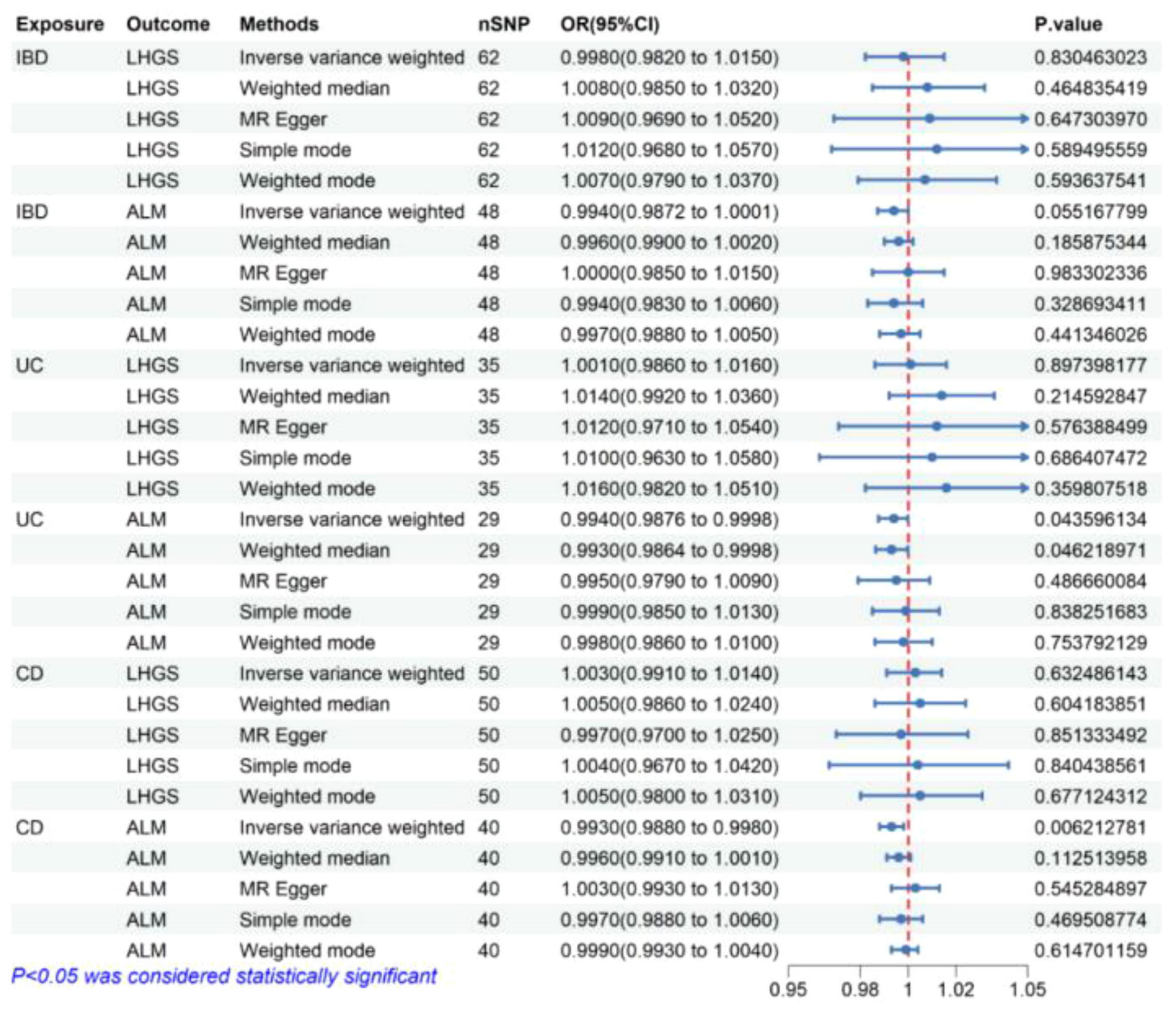
The risk association between IBD (including UC/CD) and sarcopenia-related traits in the validation set visualized in a forest plot. IBD, Inflammatory bowel disease; UC, ulcerative colitis; CD, crohn’s disease; LHGS, low hand-grip strength; ALM, appendicular lean mass; CI, confidence interval; MR Egger, Mendelian randomization-egger regression; nSNP, number of SNPs (instrumental variables); OR, odds ratio.

As is shown in **Figure 2**, the result of the IVW method in MR analysis showed that no causal association was observed between IBD, UC, and CD negatively with LHGS (all P value> 0.05).

#### 3.1.2 Causal effects of IBD on ALM

The result of the IVW method in MR analysis showed that genetically predicted UC (OR = 0.994, 95% CI 0.9876-0.9998, P = 0.044) and CD (OR = 0.993, 95% CI 0.988-0.998, P= 0.006) significantly negatively correlated with ALM. In the median weight model, causal effect of UC on ALM was consistent with the trend of the IVW model and reached statistical significance (OR = 0.993, 95% CI 0.9864-0.9998, P = 0.046), causal effect of CD on ALM showed a consistent direction but insignificant results (P> 0.05) (**Figure 2**).

### 3.2 Causal effects of Sarcopenia-related traits on IBD

#### 3.2.1 Causal effects of LHGS on IBD

**Figure 3:**
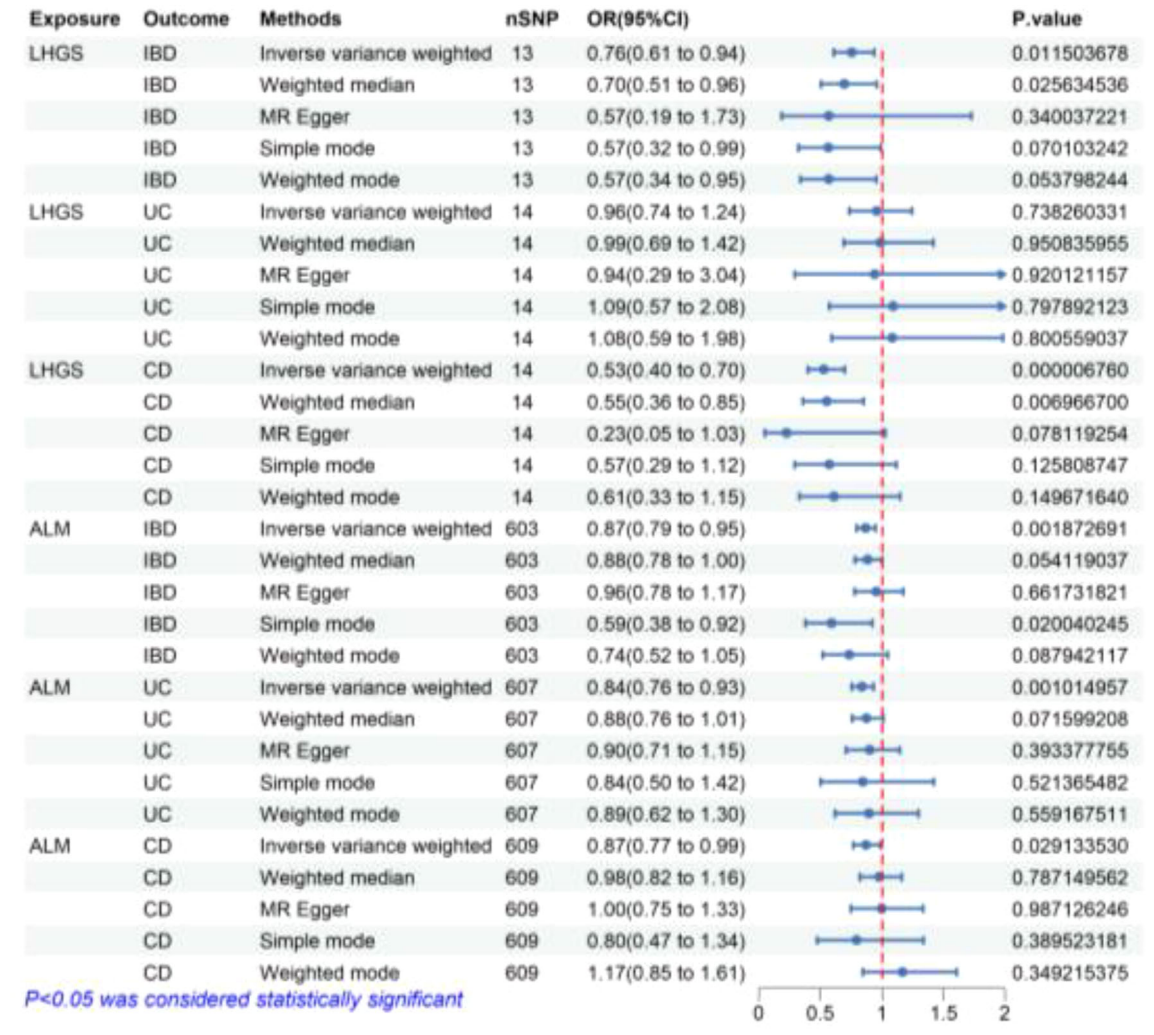
The risk association between sarcopenia-related traits and IBD (including UC/CD) in the validation set visualized in a forest plot. LHGS, low hand-grip strength; ALM, appendicular lean mass; IBD, Inflammatory bowel disease; UC, ulcerative colitis; CD, crohn’s disease; CI, confidence interval; MR Egger, Mendelian randomization-egger regression; nSNP, number of SNPs (instrumental variables); OR, odds ratio.

According to the findings presented in **Figure 3**, the IVW method in reverse MR analysis revealed a significant negative association between the genetic predisposition to LHGS and the occurrence of IBD (OR = 0.76, 95% CI 0.61-0.94, P=0.012) as well as CD (OR = 0.76, 95% CI 0.61-0.94, P=0.012). Besides, weighted median method also showed that LHGS significantly negatively correlated with IBD (OR = 0.70, 95% CI 0.51-0.96, P=0.025) and CD (OR = 0.55, 95% CI 0.36-0.85, P=0.007).

#### 3.2.2 Causal effects of ALM on IBD

Similarly, the IVW method yielded findings that indicate a negative connection between ALM and IBD and IBD subtypes, including IBD (OR = 0.87, 95% CI 0.79-0.95, P=0.002), UC (OR = 0.84, 95% CI 0.76-0.93, P=0.001) and CD (OR = 0.87, 95% CI 0.77-0.99, P=0.029). Meanwhile, simple mode method also showed that ALM significantly negatively correlated with IBD (OR = 0.59, 95% CI 0.38-0.92, P=0.020) and CD (OR = 0.55, 95% CI 0.36-0.85, P=0.007). The MR estimates from remaining different methods of assessing the causal effect between ALM and IBD related diseases did not indicate a directly causal effect.

### 3.3 Sensitivity analysis

Following a sequence of sensitivity analyses, it was determined that the IVW results were stable after implementing the MR-PRESSO correction and weighted median method (**Figures 2**, **3**). Cochrane’s Q and Q-derived p values were computed in order to evaluate the heterogeneity of our estimates. Although significant heterogeneity was detected in some of our results (**Supplementary Figure 1**), the importance of the IVW estimates remains after adjustment using a random effects model. Furthermore, in order to identify possible potential pleiotropic effects, we employed the p-value associated with the MR-Egger intercept. It is worth noting that only the causal impact of CD on ALM exhibits potential pleiotropy (P=0.029) (**Supplementary Figure 2**). Furthermore, we used the weighted mode, the simple mode, the MR-Egger, and the weighted median to evaluate the effects of genetically predicted exposures on outcomes, and the results were found to be relatively robust. In addition, the leave-one-out analysis revealed that the majority of results did not exhibit any significant alteration in the correlation when individual variation was removed (**Supplementary Figure 3**).

## 4 Discussion

IBD is a chronic condition affecting the digestive system, characterized by immune-mediated inflammation of unknown origin. It is frequently accompanied by extraintestinal symptoms, including peripheral arthritis, oral aphthous ulcers, episcleritis, and erythema nodosum (27). The incidence of IBD is steadily growing, which will result in a larger population being susceptible to problems associated with the condition in the coming decade (28). To mitigate this risk and advance care, there exists a strong requirement for enhanced risk stratification instruments that can detect potentially modifiable characteristics and provide guidance on the appropriate timing and selection of treatment options. However, there are currently limited risk stratification tools for IBD, mainly because currently identified risks do not include indicators that reflect periods of persistent inflammation (29). Individuals with IBD frequently experience compromised nutritional status, which is evident by disturbances in energy or nutrient consumption. This impairment encompasses several forms of malnutrition, such as protein-energy malnutrition, disease-associated malnutrition, deficits in micronutrients, and sarcopenia (28).

Sarcopenia, a medical disorder characterized by the decline in muscular strength and mass, can be attributed to the presence of inflammation. Sarcopenia, commonly linked to the aging process, has been shown to start at an earlier stage in life in recent study. Furthermore, this condition has been found to have a significant correlation with heightened amounts of inflammatory cytokines circulating inside the body (1). Clinical researches have also shown a higher prevalence of sarcopenia in patients with inflammation (30). In addition, over the past few years, there has been increasing evidence that sarcopenia is strongly associated with adverse clinical outcomes, including abdominal surgery (31, 32). Nardone et al. (33) demonstrated that the implementation of suitable interventions for sarcopenia is crucial, alongside achieving clinical remission, in order to enhance clinical outcomes among patients diagnosed with IBD.

Nevertheless, data to assess both IBD as a risk stratification tool for sarcopenia and sarcopenia as a risk stratification tool for IBD are heterogeneous and scarce, which also leads to an unclear understanding of the interaction between IBD and sarcopenia (34). The current study by Campbell et al. found that 24% of patients with CD were identified as sarcopenia (35). Fatani et al. (14) also found that the overall prevalence of sarcopenia in IBD was noted to be 42%, with a higher prevalence in CD (57%). These prevalence rates were significantly higher as compared with patients without IBD. However, Lee et al. (36) demonstrated that sarcopenia was not statistically significant in the prognosis of CD. But unfortunately, given the retrospective nature of prior studies, observational studies or meta-analyses don’t have enough evidence to prove a causal relationship between IBD and sarcopenia (37).

In this study, we employed the largest available GWAS data from the IIBDGC large-scale and a sarcopenia meta-analysis to examine the potential bidirectional causal relationship between IBDs and sarcopenia-related traits susceptibility. According to our forward MR Analysis, both UC and CD were causally related with reduced ALM (OR=0.99, 95% CI: 0.99 - 1.00; OR=0.99, 95% CI: 0.99 - 1.00, respectively). Notable, reverse MR analysis also showed a causal association between LHGS and IBD (OR=0.76, 95% CI: 0.61- 0.94) or CD (OR=0.53, 95%CI: 0.40 - 0.70). Meantime, the increase in ALM was causally related with a reduced risk of IBD (OR=0.87, 95%CI: 0.79-0.95) and IBD subtype (OR=0.84, 95%CI: 0.76 - 0.93 for UC; OR=0.87, 95% CI: 0.77-0.99 for CD).

The primary finding of our MR study was a significant causal association between sarcopenia and IBDs. This result is similar to previous findings that sarcopenia is substantially associated with an increased risk of adverse outcomes for IBD such as treatment failure and postoperative complications (38–40). Although only UC and CD were causally associated with decreased ALM, it was not associated with decreased grip strength. However, it is important to note that persistent chronic inflammation and malnutrition collectively contribute to the susceptibility of individuals with IBD to the onset of sarcopenia (28). This also highlights the potential of UC and CD as risk stratification tools for sarcopenia.

As a study using SNP as a tool to explore the causal relationship between IBDs and sarcopenia. Our study offers several advantages. First, we showed that IBD was found to have a significant negatively effect on ALM and not causally associated with decreased grip strength. Second, the MR Study is regarded as a natural randomized controlled trial study, and its findings exhibit a higher level of robustness compared to earlier observational studies. Furthermore, we performed some sensitivity analyses to assess the robustness of the results. Finally, the research was constrained to a European demographic in order to mitigate any biases in the selection of the population, while also excluding confounding factors such as age, physical activity, socioeconomic status, and poor diet.

Despite the progress made in enhancing our present comprehension of the correlation between IBD and sarcopenia, the study still has some shortcomings. Firstly, although the fact that the F statistic suggests that the biases due to feeble IVs can be disregarded, the pathway from exposure to outcome is extremely complex, and some results are heterogeneous, so caution should be exercised when interpreting these results. Secondly, we only included LHGS and ALM in sarcopenia-related traits, and other important indicators such as walking speed were not included. Thirdly, all GWAS data in this study came primarily from European populations. While such measures can be effective in reducing bias from different populations, the study’s conclusions may not be directly transferable to other ethnic groups. Fourthly, the causal relationship analyzed by MR is derived from the genetic level, and the body contains a variety of complex biological pathways, so the potential causal relationship is not a definite causal relationship. Finally, the statistical efficacy of MR Analysis is relatively weak due to the limited sample size and number of SNP of IBD, and a larger and updated IBD database may be needed to confirm causality.

## 5 Conclusion

In conclusion, this study suggests that UC and CD disease are causally associated with reduced ALM. In turn, higher hand grip strength reduced the risk of IBD and CD, and higher ALM was associated with a reduced risk of IBD (including UC and CD subtypes). These findings suggest that intervention with sarcopenia may help prevent adverse outcomes in patients with IBD.

## Data availability statement

The datasets presented in this study can be found in online repositories. The names of the repository/repositories and accession number(s) can be found in the article/**Supplementary Material**.

## Ethics statement

All datasets provided in GWAS have been approved by the relevant ethics committees. The studies were conducted in accordance with the local legislation and institutional requirements. Written informed consent for participation in this study was provided by the participants’ legal guardians/next of kin.

## Author contributions

ZS: Writing – original draft. GL: Writing – review & editing, Data curation, Conceptualization. JX: Writing – review & editing, Project administration, Data curation. XZ: Writing – review & editing, Supervision. HW: Writing – review & editing, Project administration. GW: Writing – original draft, Visualization. JJ: Writing – original draft, Visualization, Funding acquisition.
