# Supplementary material for: The relationship between inflammatory bowel disease and sarcopenia-related traits: a bidirectional two-sample mendelian randomization study
Source: Front Endocrinol (Lausanne). 2024 Jul 12;15:1402551. doi: 10.3389/fendo.2024.1402551 (PMC11272465; doi:10.3389/fendo.2024.1402551)
Supplement: Supplementary file 1 [file DataSheet_1.zip › Supplementary Image 2.DOCX]

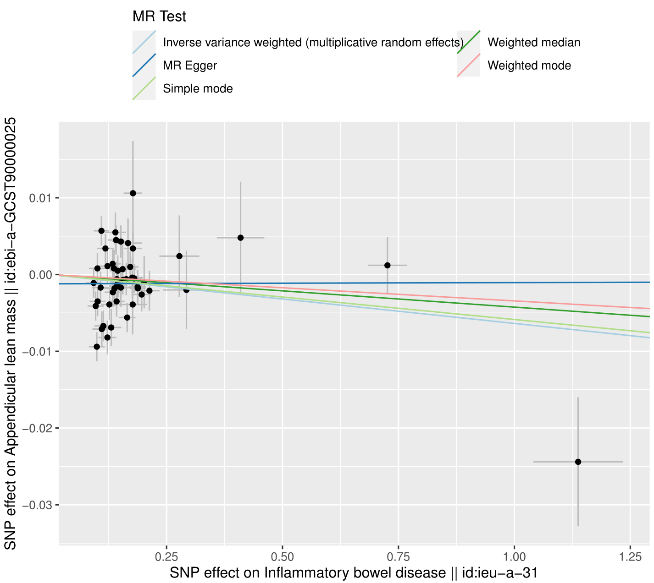

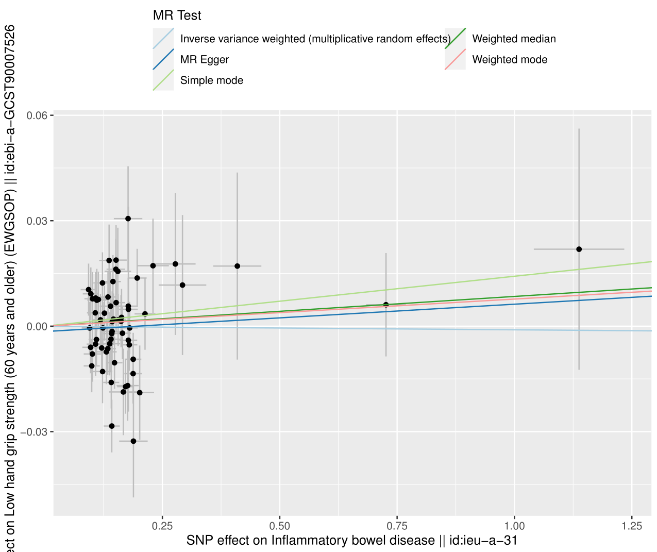

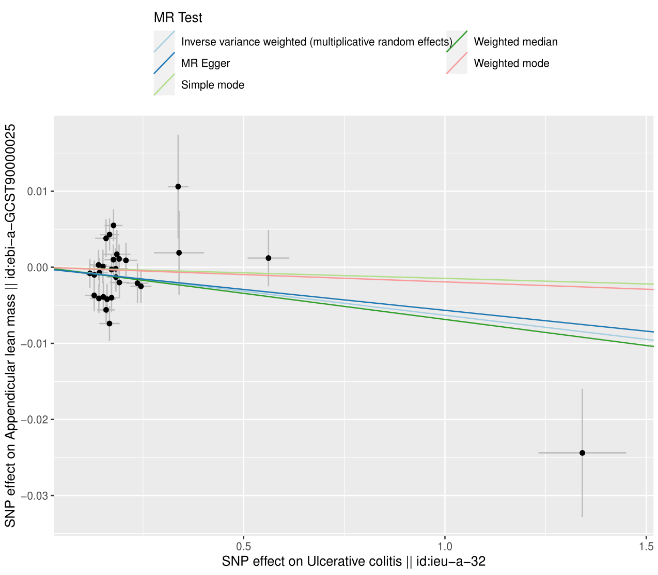

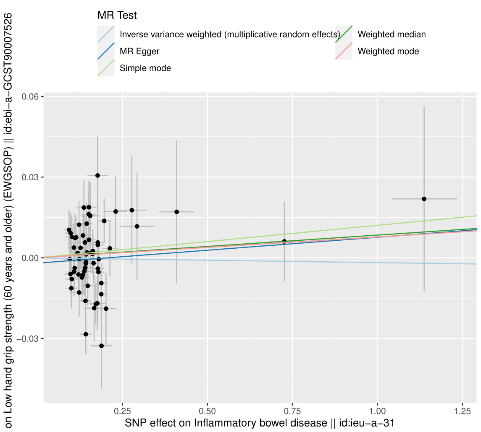
(a)Causal effect of IBD on LHGS (b)Causal effect of IBD on ALM


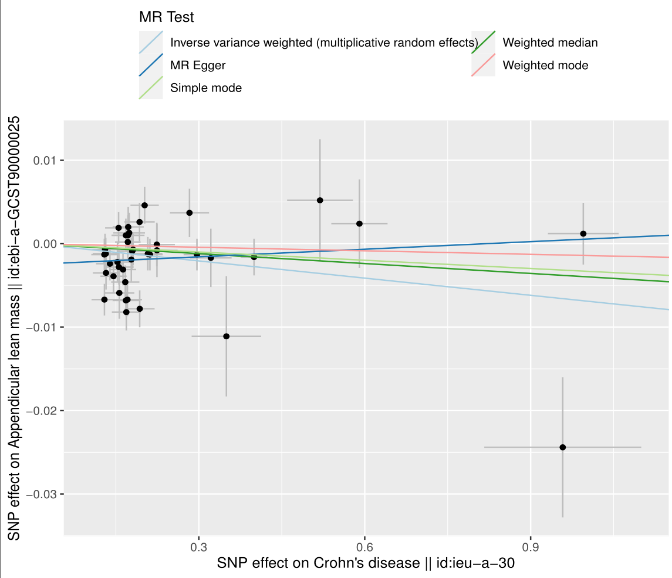

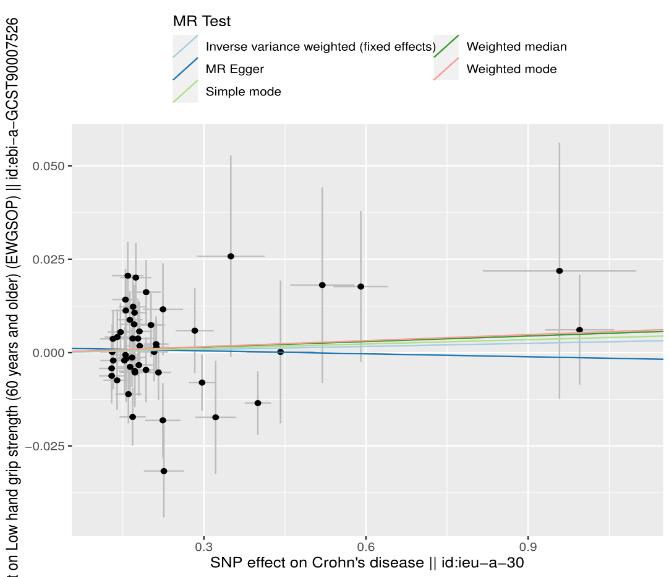
(c)Causal effect of UC on LHGS (d)Causal effect of UC on ALM

(e)Causal effect of CD on LHGS (f)Causal effect of CD on ALM


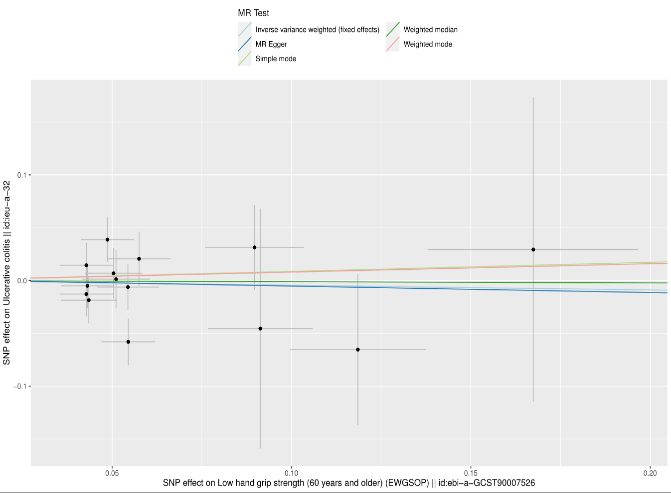

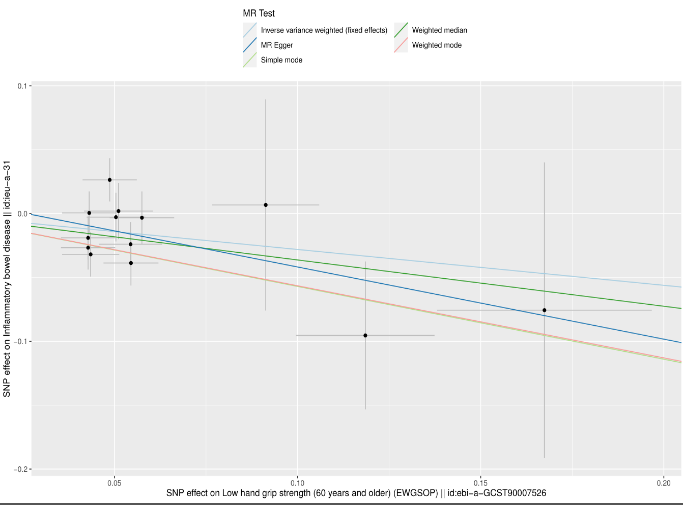


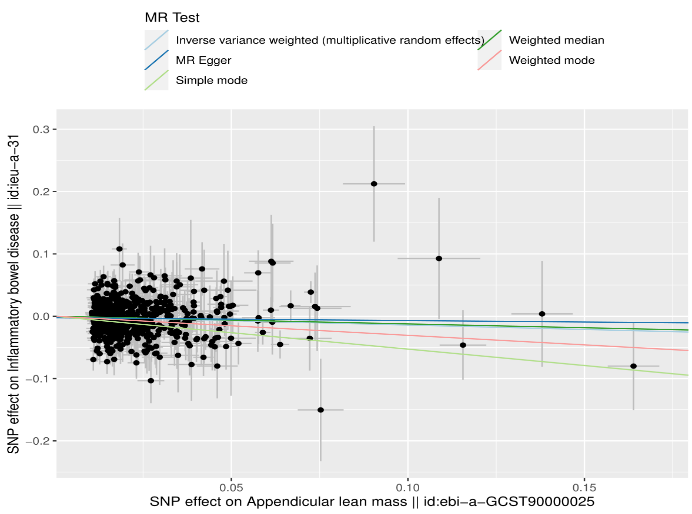

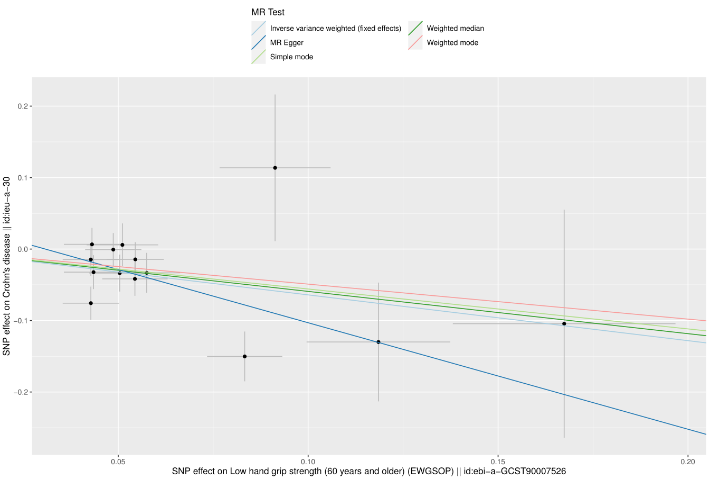
(g)Causal effect of LHGS on IBD (h)Causal effect of LHGS on UC


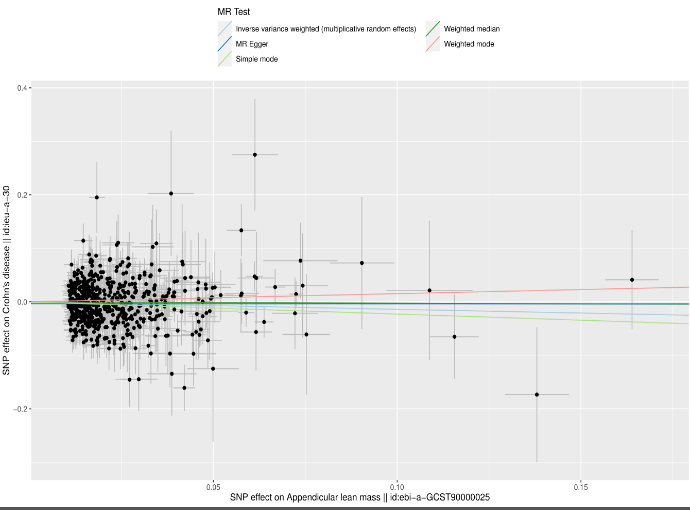

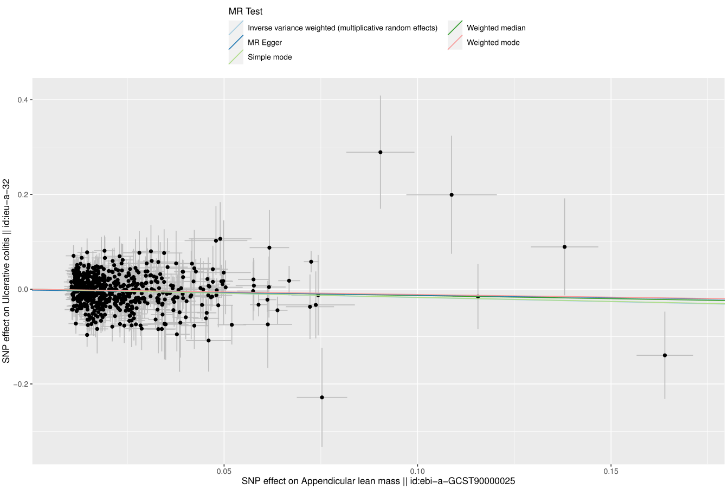
(i)Causal effect of LHGS on CD (j)Causal effect of ALM on IBD

(k)Causal effect of ALM on UC (l)Causal effect of ALM on CD

**Supplementary figure 2** Scatterplots and causal effects estimated. (a) causal effect of inflammatory bowel disease (IBD) on Low hand grip strength (LHGS); (b) causal effect of IBD on appendicular lean mass (ALM); (c) causal effect of ulcerative colitis (UC) on LHGS; (d) causal effect of ulcerative colitis (UC) on ALM; (e) causal effect of Crohn's disease (CD) on LHGS; (f) causal effect of CD on ALM; (g) causal effect of LHGS on IBD; (h) causal effect of LHGS on UC; (i) causal effect of LHGS on CD; (j) causal effect of ALM on IBD; (k) causal effect of ALM on UC; (l) causal effect of ALM on CD.
